# Robot-Assisted TKA for Varus Knees: Post Hoc Exploratory Analysis of Alignment Strategy and Deformity Severity

**DOI:** 10.3390/jcm15124515

**Published:** 2026-06-11

**Authors:** Alexey Vladimirovich Lychagin, Andrey Anatolyevich Gritsyuk, Mikhail Pavlovich Elizarov, Andrey Andreevich Gritsyuk, Konstantin Khadisovich Tomboidi, Manuchehr Mukhsidinovich Khalimov, Eugene Borisovich Kalinsky, Nahum Rosenberg

**Affiliations:** Department of Traumatology, Orthopedics, and Disaster Surgery, I.M. Sechenov First Moscow State Medical University (Sechenov University), Trubetskaya Str., 8-2, 119991 Moscow, Russia; clinic@travma.moscow (A.V.L.); elizarovm07@gmail.com (M.P.E.); andrewgritsru@gmail.com (A.A.G.J.); tomboidi@mail.ru (K.K.T.); manuchehrhalimov1@gmail.com (M.M.K.); kalinsky_e_b@staff.sechenov.ru (E.B.K.); nahumrosenberg@sheltagen.com (N.R.)

**Keywords:** robot-assisted total knee arthroplasty, mechanical alignment, restricted kinematic alignment, varus knee, coronal correction, patient-reported outcomes

## Abstract

**Background:** Robot-assisted total knee arthroplasty (raTKA) improves the precision of component positioning and coronal alignment restoration, but it remains uncertain whether that technical accuracy modifies the clinical effect of alignment strategy in different varus phenotypes. The present report evaluates alignment strategy and correction magnitude, explicitly as a post hoc exploratory deformity-subgroup analysis within a randomized raTKA cohort. **Methods:** This single-center, open-label, randomized study enrolled 296 patients with varus knee osteoarthritis who underwent raTKA between 2023 and 2025 using either mechanical alignment (MA; *n* = 149) or limited/restricted kinematic alignment (lim.-KA; *n* = 147). The parent randomized comparison was conducted at the whole-cohort level; the deformity-based subgroups reported here were defined after the whole-cohort analysis and are therefore post hoc and exploratory. Patients were stratified according to preoperative varus severity into a mild-deformity subgroup (≤10°; lim.-KA-I *n* = 99, MA-I *n* = 102) and a moderate-deformity subgroup (11–20°; lim.-KA-II *n* = 48, MA-II *n* = 47). Outcomes included hip–knee–ankle angle (HKA), correction angle, range of motion (ROM), visual analog scale (VAS; 0–10 points), Knee Society Score (KSS; knee and function), Oxford Knee Score (OKS), and Forgotten Joint Score-12 (FJS-12) over 12 months. Estimates are presented with 95% confidence intervals where applicable. Because multiple post hoc subgroup comparisons were performed without formal multiplicity adjustment, *p*-values are interpreted descriptively and in conjunction with effect sizes and 95% confidence intervals. **Results:** The primary whole-cohort randomized comparison did not demonstrate an overall between-group advantage of either alignment strategy. The post hoc moderate-varus subgroup showed favorable unadjusted 12-month differences for lim.-KA versus MA in KSS-knee (+6.8 points; 95% CI 5.3 to 8.3; nominal *p* < 0.001), KSS-function (+4.0 points; 95% CI 2.7 to 5.2; nominal *p* < 0.001), OKS (+6.4 points; 95% CI 4.5 to 8.3; nominal *p* < 0.001), and FJS-12 (+11.3 points; 95% CI 9.4 to 13.1; nominal *p* < 0.001). In contrast, ROM favored MA rather than lim.-KA in the moderate-varus subgroup (−11.8°; 95% CI −16.6 to −7.0; nominal *p* < 0.001), indicating greater 12-month ROM after MA, and VAS pain, reported on a 0–10 scale, did not support a lim.-KA pain advantage (+0.26 points; 95% CI 0.05 to 0.48; higher scores indicate worse pain; nominal *p* = 0.018). Exploratory, unadjusted, post hoc 12-month alignment-by-deformity interaction terms were significant for ROM, KSS-knee, KSS-function, OKS, and FJS-12, but not for VAS. Because multiple post hoc comparisons were performed without formal multiplicity adjustment, the results are interpreted descriptively, along with effect sizes and confidence intervals. **Conclusions:** The primary randomized comparison did not demonstrate a clinical advantage of lim.-KA over MA in the whole cohort. In post hoc exploratory analyses, mild varus deformity was associated with outcomes broadly similar to those after both alignment strategies. In the moderate-varus subgroup, patient-level analyses suggested a possible phenotype-dependent signal for KSS-knee, KSS-function, OKS, and FJS-12 after lim.-KA, whereas ROM favored MA, and VAS pain did not support a lim.-KA pain advantage. These subgroup findings should be interpreted separately from the primary randomized result, considered hypothesis-generating only, and not used in isolation to change clinical practice without prospective confirmation.

## 1. Introduction

Total knee arthroplasty (TKA) is an established treatment for end-stage knee osteoarthritis, and its use is projected to continue increasing in the coming decades [1]. Mechanical alignment (MA) has traditionally been regarded as the reference coronal alignment strategy in TKA [2]. Despite the durability and reproducibility of this approach, patient dissatisfaction persists in a meaningful minority of cases after otherwise technically successful surgery [3,4]. This discrepancy between technical success and subjective outcome has sustained interest in individualized alignment philosophies that may better reflect constitutional anatomy, joint-line orientation, and soft-tissue behavior [2,5].

The clinically important unresolved question is not simply whether individualized alignment can reproduce native anatomy, but whether alignment strategy interacts with deformity severity and correction magnitude. This question is particularly relevant in varus knees, where correction to a neutral mechanical axis may require different degrees of alteration in ligament tension, joint-line orientation, and gap symmetry depending on the preoperative phenotype [2,3,4,5].

Kinematic alignment (KA) and related strategies aim to restore patient-specific or pre-arthritic limb and joint-line geometry rather than applying the same neutral target to every knee [5,6]. Restricted or limited kinematic alignment (lim.-KA) is a bounded version of this concept that attempts to preserve individualized alignment while constraining the final limb and component positions within predefined safety limits [7,8,9].

Varus osteoarthritis is morphologically heterogeneous rather than a single deformity pattern. Increasing varus deformity is associated with differences in medial and lateral soft-tissue behavior, constitutional limb shape, and joint-space asymmetry, all of which may influence the clinical effect of a given alignment target [10,11,12].

Robot-assisted TKA (raTKA) provides a useful platform for evaluating this issue because robotic planning and execution can improve the precision with which different alignment targets are delivered [13,14,15]. However, robotic precision alone has not consistently translated into large short-term clinical advantages over conventional instrumentation, suggesting that the clinical effect of robotics may depend on the alignment plan used and the phenotype to which it is applied [16,17].

Accordingly, this study adds to the published reports on robotic TKA alignment by separating two questions: whether the parent randomized comparison showed an overall group effect, and whether a post hoc exploratory deformity-stratified analysis suggests that the alignment strategy may behave differently in mild- versus moderate-varus knees. The latter question is hypothesis-generating and requires cautious interpretation.

The present study was designed in this context. Although the overall randomized comparison between MA and lim.-KA did not demonstrate a significant whole-cohort difference, varus knees are phenotypically heterogeneous, and correction magnitude differs substantially between mild and moderate deformity. Subgroup analyses based on preoperative varus severity may therefore yield clinically useful information, provided they are interpreted cautiously. The aim of this study was to compare the short-term radiographic, clinical, and functional outcomes of raTKA performed with MA versus lim.-KA in patients with varus knee osteoarthritis and to explore whether these outcomes differed by preoperative varus severity and the magnitude of coronal correction. The exploratory subgroup hypothesis was that the association between alignment strategy and postoperative outcome might differ by deformity severity and correction magnitude; however, confirmation of a true phenotype-dependent effect would require a prespecified alignment-by-deformity interaction analysis.

## 2. Materials and Methods

### 2.1. Study Design and Ethics

This was a single-center, open-label comparative clinical study of robot-assisted primary total knee arthroplasty for varus knee osteoarthritis conducted between 2023 and 2025 at a tertiary academic medical center. The study was performed within a prospectively registered clinical protocol (ClinicalTrials.gov identifier NCT05750784) and was approved by the Sechenov University Ethics Committee (No. 25-22, 8 December 2022). The primary comparison was between mechanical alignment and limited/restricted kinematic alignment, whereas the deformity-stratified subgroup analysis reported in this manuscript was retrospective/post hoc and should be interpreted as exploratory. Trial reporting included an explicit description of participant flow, randomization, allocation concealment, and analysis of populations.

The ClinicalTrials.gov record for NCT05750784 defines CT-based implant-position assessments as the protocol-level primary outcomes and lists OKS as a secondary outcome. The present manuscript is not the primary registered imaging-outcome report. It is a secondary clinical-outcome analysis of the available randomized patient-level cohort, focused on alignment strategy, deformity severity, and 12-month clinical recovery. For this reason, 12-month OKS is reported as the principal clinical endpoint of the present secondary analysis, while remaining a secondary outcome in the registered parent protocol. The enrolment difference reflects the distinction between the registry’s estimated enrolment target and the available analytic cohort from the broader institutional study period. After application of the same eligibility framework, 296 randomized patients were available for the present patient-level clinical analysis.

### 2.2. Eligibility Criteria

Eligible participants were adults aged 18 years or older with symptomatic varus knee osteoarthritis scheduled for primary TKA, a coronal varus deformity of up to 20°, American Society of Anesthesiologists class III or lower, body mass index below 35 kg/m^2^, and anticipated availability for 12 months of follow-up. Patients were excluded if they had valgus alignment, severe varus deformity greater than 20°, collateral ligament insufficiency requiring constrained implants, major bone loss requiring augmentation, impairment of contralateral limb support, prior major reconstructive surgery around the index knee, or post-traumatic osteoarthritis. Patients who withdrew consent or failed to adhere to the follow-up schedule were not included in the analytic dataset.

### 2.3. Randomization and Analytic Framework

The present manuscript reports a secondary exploratory analysis conducted within a broader ethics-approved clinical study. According to the parent study protocol, eligible patients were randomized in a 1:1 ratio to mechanical alignment (MA) or limited/restricted kinematic alignment (lim.-KA) using a computer-generated randomization sequence. No blocking or stratification was used. The randomization sequence was stored in a computerized database and was generated without prior involvement of the operating surgeons. Patient enrolment was performed by the surgical team after confirmation of eligibility and provision of informed consent.

Allocation concealment was maintained through a central computerized randomization process, with group assignments prepared in sequentially numbered, opaque, sealed envelopes by staff members independent of patient enrolment and surgery. Group assignment was revealed only after eligibility confirmation and informed consent and was then incorporated into the robotic preoperative plan before surgery. Because of the nature of the intervention, surgeons could not be blinded to the allocated alignment strategy. However, clinical outcome assessors, radiographic assessors, and statistical analysts were blinded to group allocation. Blinding was maintained by providing assessors with coded clinical forms and radiographs that did not contain the randomized alignment assignment, robotic plan, operative alignment target, or group label; the statistical dataset used coded group identifiers until the prespecified analyses were completed. Accordingly, 149 patients underwent MA and 147 underwent lim.-KA. Because the whole-cohort comparison did not demonstrate a significant between-group difference, the deformity-stratified subgroup analyses presented in this manuscript are interpreted as post hoc, exploratory, and hypothesis-generating rather than confirmatory.

Because the study was open-label, surgeons were necessarily aware of the assigned alignment strategy during robotic planning and execution. This design may introduce performance bias and expectation-related bias, particularly for patient-reported outcomes, even though outcome assessors and statistical analysts were blinded to group allocation.

The analyzed cohort was generated strictly from the predefined inclusion and exclusion criteria of the parent study. The dataset available for this secondary analysis did not contain the number of patients assessed before enrolment or the reasons for non-inclusion before randomization. Consequently, the full pre-randomization screening pathway could not be reconstructed. This limits assessment of representativeness and external generalizability, although transparent reporting of eligibility criteria and participant-selection methods remains an accepted approach for defining the analytic population [18].

### 2.4. Subgroup Definition

**Figure 1:**
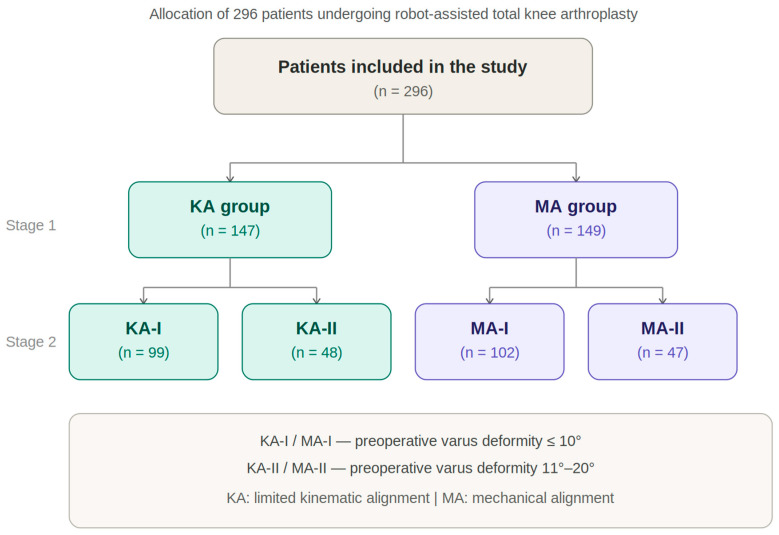
**Allocation of patients scheduled for raTKA.**

The dataset was stratified post hoc by severity of preoperative varus deformity. The mild-varus subgroup (≤10° varus) comprised 99 lim.-KA patients (lim.-KA-I) and 102 MA patients (MA-I). The moderate-varus subgroup (11–20° varus) comprised 48 lim.-KA patients (lim.-KA-II) and 47 MA patients (MA-II). This classification corresponded closely to achieved correction magnitude: approximately 4–6° in the mild-varus subgroup and approximately 11–13° in the moderate-varus subgroup. For clinical interpretation, these cutoffs should be regarded as operational research definitions rather than validated indications for selecting an alignment strategy. Patients with preoperative varus deformity >10° and an achieved correction of approximately ≥10° were classified as having a higher-correction phenotype. Patients with preoperative varus deformity ≤10° and an achieved correction of approximately 4–6° were classified as having a lower-correction phenotype. These deformity strata were not used for treatment allocation and were not prespecified as confirmatory analytic subgroups. They were examined only after the negative whole-cohort comparison. Accordingly, all subgroup results should be interpreted as exploratory, descriptive, and hypothesis-generating (Figure 1).

### 2.5. Radiographic Assessment

Coronal alignment was assessed using the hip–knee–ankle (HKA) angle measured on standing full-length weight-bearing radiographs according to established methods. The HKA is defined as the angle between the mechanical axis of the femur and that of the tibia on a full-length hip–knee–ankle radiograph and is widely used to evaluate coronal lower-limb alignment after total knee arthroplasty [19]. Preoperative HKA was recorded before surgery, and postoperative HKA was recorded at the 3-month follow-up visit. The correction angle was defined as the arithmetic difference between the preoperative and postoperative HKA values, expressed in degrees. This variable was used not only as a radiographic measure of alignment change but also as a clinically relevant indicator of the magnitude of coronal correction required by the selected alignment strategy.

### 2.6. Surgical Technique and Implant System

All procedures were performed using the CUVIS-joint CJ-150 robotic system (Curexo, Inc., Seoul, Republic of Korea), and a cemented NexGen PS-Flex fixed-bearing knee implant (Zimmer, Inc., Warsaw, IN, USA) was used in all cases. The robotic platform is a CT-based active system that allows preoperative three-dimensional planning, intraoperative registration, optical tracking, and robotic execution of planned femoral and tibial bone preparation under surgeon supervision. All procedures were undertaken by experienced arthroplasty surgeons familiar with robotic workflow and with both alignment strategies.

For MA, the surgical plan targeted restoration of a neutral mechanical axis, with femoral and tibial components positioned perpendicular to the mechanical axes of the respective bones. For lim.-KA, the plan aimed to preserve more native joint-line orientation and constitutional alignment while restricting both individual resections and final HKA within accepted safety limits consistent with previously described restricted-kinematic principles [7,8,9]. In practical terms, lim.-KA allowed a bounded residual varus phenotype rather than obligate correction to neutral. The present analysis, therefore, evaluates whether the consequences of choosing a neutral mechanical target versus a bounded residual-varus target become more clinically apparent once correction exceeds roughly 10°; this threshold is exploratory and must not be used as an independent indication for lim.-KA without prospective validation. In both groups, the robotic workflow enabled stepwise assessment of alignment targets, extension and flexion gap behavior, and component position before final bone preparation.

The numerical coronal boundary applied for lim.-KA in this varus cohort was a final limb-alignment plan of HKA 177–180° (180° = neutral), corresponding to neutral alignment to no more than 3° of residual varus. A patient-specific joint-line orientation was accepted only if this limb-level boundary was preserved. The study did not apply separate fixed Lateral Distal Femoral Angle, Medial Proximal Tibial Angle, femoral-flexion, or tibial-slope cut-offs as analytic boundaries; individual component positions were adjusted within the robotic plan to respect the limb-level HKA boundary, implant-specific planning constraints, and intraoperative soft-tissue balance. If the initial patient-specific plan exceeded the HKA boundary, the plan was adjusted toward the nearest accepted boundary while preserving balance where possible.

### 2.7. Clinical Outcomes

The principal clinical outcomes were range of motion (ROM), visual analog scale (VAS) pain score, Knee Society Score (KSS) knee and function subscales, Oxford Knee Score (OKS), and Forgotten Joint Score-12 (FJS-12). VAS pain is reported consistently on a 0–10-point scale throughout the manuscript; values recorded on the original 0–100 scale were converted by dividing by 10. ROM, VAS, KSS, and OKS were recorded preoperatively, immediately postoperatively, and at 3, 6, and 12 months. FJS-12 was recorded at 3, 6, and 12 months. These endpoints were selected to capture complementary dimensions of recovery: pain intensity, objective motion, clinician-derived function, patient-reported function, and joint awareness after arthroplasty. Together, they were intended to reflect not only whether the knee was stable and mobile after surgery, but also whether the patient found the reconstruction comfortable, functional, and integrated into daily activities.

For the present secondary clinical-outcome manuscript, the principal clinical endpoint was the Oxford Knee Score (OKS) at 12 months after surgery. Other clinical outcomes in the present analysis included FJS-12, ROM, VAS pain score, KSS knee and function subscales, postoperative hip–knee–ankle (HKA) angle, and correction angle. Serial changes in ROM, VAS, KSS, OKS, and FJS-12 were evaluated over the available follow-up intervals.

### 2.8. Statistical Analysis

Continuous variables are presented as mean ± standard deviation with 95% confidence intervals (CIs) for the group means. All group estimates, 95% confidence intervals, mean differences, and model-based estimates were calculated directly from individual patient-level observations, using the final verified study-group coding. Descriptive between-group comparisons were performed using the Mann–Whitney U test to maintain consistency with the original tabular analysis. In addition, exploratory, unadjusted, post hoc 12-month linear regression models were used to test alignment strategy, deformity severity, and the alignment-by-deformity interaction for ROM, VAS, KSS-knee, KSS-function, OKS, and FJS-12.

Multiplicity was explicitly considered in interpretation. The study includes repeated time-point-specific comparisons across several correlated outcomes and two post hoc deformity strata, but no formal family-wise-error or false-discovery-rate correction was applied. Therefore, nominal *p*-values are reported as descriptive measures only and should not be interpreted as confirmatory evidence. To address the baseline VAS imbalance in the moderate-varus subgroup, an exploratory baseline-adjusted ANCOVA model was performed for 12-month VAS on the 0–10 scale. A repeated-measures sensitivity analysis was also performed using exploratory, unadjusted, post hoc generalized estimating equations with patient-level clustering, time, alignment strategy, deformity severity, and their interactions [20]. The primary interpretive emphasis remains on effect sizes, 95% CIs, consistency across related outcomes, and established clinical relevance thresholds.

GEE was selected instead of a mixed-effects model because this sensitivity analysis was intended to estimate population-average differences in longitudinal outcome trajectories rather than subject-specific random effects. This approach was appropriate for the present post hoc analysis because it accounts for within-patient correlation across repeated measurements without requiring specification of a random-effect structure in relatively small deformity subgroups.

A formal outcome-specific a priori sample-size calculation was not available for the present post hoc subgroup analysis. The total cohort included 296 patients, with near-equal allocation between the two alignment groups (MA, *n* = 149; lim.-KA, *n* = 147). After post hoc stratification by deformity severity, subgroup sizes remained closely balanced but smaller (mild varus: lim.-KA-I, *n* = 99; MA-I, *n* = 102; moderate varus: lim.-KA-II, *n* = 48; MA-II, *n* = 47). Exploratory detectable-difference estimates were calculated to clarify the approximate sensitivity of the subgroup analyses. Using the observed 12-month standard deviations, two-sample comparisons with 80% power and a two-sided alpha of 0.05 would be expected to detect differences of approximately 5.1° for ROM, 0.18 points for VAS on the 0–10 scale, 1.8 points for KSS-knee, 1.0 point for KSS-function, 0.4 points for OKS, and 2.1 points for FJS-12 in the mild-varus subgroup. In the moderate-varus subgroup, the corresponding detectable differences were approximately 6.7° for ROM, 0.31 points for VAS on the 0–10 scale, 2.1 points for KSS-knee, 1.7 points for KSS-function, 2.7 points for OKS, and 2.6 points for FJS-12. These thresholds were larger in the moderate-varus subgroup for some outcomes because of the smaller sample size and, in some cases, greater variability. These calculations are approximate and exploratory. They indicate that the subgroup analyses were better suited to detecting moderate-to-large differences than small differences, particularly for ROM and VAS in the moderate-varus subgroup. Therefore, the subgroup findings should be interpreted as hypothesis-generating rather than confirmatory.

All statistical analyses were performed using SPSS Statistics 22.0 (SPSS Inc., Chicago, IL, USA).

## 3. Results

### 3.1. Study Cohort and Baseline Characteristics

Participant flow is summarized in Figure 1. A total of 296 randomized patients were reported to have received the allocated intervention and to have completed the 12-month follow-up period. No loss to follow-up was reported at 12 months. The analysis, therefore, used the complete available randomized cohort without imputation. There were no protocol deviations leading to exclusion from the 12-month analytic dataset. The deformity-stratified subgroups were well matched for age, body mass index, preoperative HKA, and preoperative ROM. In the mild-varus stratum, mean age was 66.84 ± 7.05 years in lim.-KA-I and 66.50 ± 7.71 years in MA-I, with corresponding body mass index values of 30.23 ± 3.52 and 30.23 ± 3.97 kg/m^2^. Mean preoperative HKA was 172.69 ± 1.03 degrees in lim.-KA-I and 172.80 ± 1.06 degrees in MA-I, while preoperative ROM was 85.4 ± 4.7 degrees and 85.5 ± 4.7 degrees, respectively.

In the moderate-varus subgroup, mean age was 67.65 ± 7.41 years in lim.-KA-II and 66.68 ± 7.00 years in MA-II, with body mass index values of 29.98 ± 3.73 and 30.93 ± 3.09 kg/m^2^, respectively. Preoperative HKA was 165.73 ± 2.96° in lim.-KA-II and 165.68 ± 2.75° in MA-II, while preoperative ROM was 84.8 ± 5.5° and 85.7 ± 3.9°. These data indicate that within matched deformity strata, the alignment groups began from similar baseline deformity severity and motion status. One exception was pain in the moderate-varus subgroup: preoperative VAS was 6.46 ± 1.37 points in the lim.-KA-II group and 5.78 ± 1.49 points in the MA-II group, a difference that reached statistical significance (*p* = 0.026). This imbalance is relevant when interpreting later pain comparisons. Baseline characteristics are summarized in Table 1.

### 3.2. Radiographic Outcomes

Postoperative HKA values were consistent with the intended alignment philosophy. In the mild-varus subgroup, mean postoperative HKA was 176.88 ± 1.10° in lim.-KA-I and 179.28 ± 1.00° in MA-I. In the moderate-varus subgroup, mean postoperative HKA was 176.73 ± 1.05° in lim.-KA-II and 179.09 ± 1.12° in MA-II. These findings confirm that lim.-KA preserved a bounded residual varus phenotype, whereas MA restored a more neutral postoperative axis.

The achieved correction angle similarly reflected both baseline deformity and surgical target. In the mild-varus subgroup, the mean correction angle was 4.20 ± 1.44° in lim.-KA-I and 6.48 ± 1.48° in MA-I. In the moderate-varus subgroup, the correction angle increased to 11.06 ± 3.10° in lim.-KA-II and 13.30 ± 2.87° in MA-II. Thus, within both deformity strata, MA required more correction toward neutrality than lim.-KA, and the absolute difference in correction magnitude became more substantial as preoperative varus increased. Postoperative HKA and correction magnitude are summarized in Table 2.

### 3.3. Range of Motion

In the mild-varus subgroup, ROM improved progressively after surgery in both alignment groups, with no clinically meaningful between-group difference. At 12 months, ROM was 119.4 ± 13.2 degrees (95% CI 116.8 to 122.0) in lim.-KA-I and 116.4 ± 12.4 degrees (95% CI 114.0 to 118.8) in MA-I (*p* = 0.081), indicating only a small numerical difference.

In the moderate-varus subgroup, the patient-level dataset showed higher ROM after MA than after lim.-KA. Immediately after surgery, ROM was 96.0 ± 7.0 degrees (95% CI 94.0 to 98.0) in lim.-KA-II and 100.2 ± 7.0 degrees (95% CI 98.2 to 102.2) in MA-II (*p* = 0.006). This pattern persisted at 3 months (107.7 ± 9.7 versus 114.6 ± 6.5 degrees; *p* < 0.001), 6 months (115.3 ± 13.1 versus 124.9 ± 8.8 degrees; *p* < 0.001), and 12 months (115.8 ± 14.5 versus 127.6 ± 8.1 degrees; *p* < 0.001). Therefore, ROM should not be interpreted as supporting a lim.-KA advantage in the moderate-varus subgroup (Table 3).

### 3.4. Pain Outcomes

Pain improved substantially over time in all subgroups. In the mild-varus subgroup, 12-month VAS scores on the 0–10 scale were low in both groups: 0.38 ± 0.50 (95% CI 0.29 to 0.48) in lim.-KA-I and 0.26 ± 0.40 (95% CI 0.18 to 0.34) in MA-I (*p* = 0.447). This small difference did not indicate a consistent clinical advantage in pain for either alignment strategy.

In the moderate-varus subgroup, baseline VAS was higher in lim.-KA-II than in MA-II, and postoperative pain outcomes did not support a causal pain advantage for lim.-KA. At 12 months, VAS on the 0–10 scale was 0.67 ± 0.55 (95% CI 0.52 to 0.83) in lim.-KA-II and 0.41 ± 0.52 (95% CI 0.26 to 0.56) in MA-II (*p* = 0.059). After adjustment for baseline VAS, 12-month pain remained numerically higher in the lim.-KA-II group than in the MA-II group. The adjusted lim.-KA minus MA difference was +0.23 points on the 0–10 VAS (95% CI 0.005 to 0.45; *p* = 0.045), where higher values indicate worse pain. This small adjusted difference does not support a pain-relief advantage of lim.-KA in the moderate-varus subgroup. Thus, the pain analysis should be interpreted cautiously and does not support a stand-alone pain-relief benefit of lim.-KA (Table 3).

### 3.5. Knee Society Score

In the mild-varus subgroup, the KSS domains showed no clinically consistent separation between alignment strategies. At 12 months, KSS-knee was 86.3 ± 4.5 (95% CI 85.4 to 87.2) in lim.-KA-I and 87.7 ± 4.7 (95% CI 86.7 to 88.6) in MA-I (*p* = 0.032), whereas KSS-function was 92.5 ± 2.7 and 93.0 ± 2.4, respectively (*p* = 0.248). The small nominal KSS-knee difference should be interpreted descriptively in the context of multiplicity and limited clinical separation.

In the moderate-varus subgroup, KSS values were numerically higher after lim.-KA across follow-up. At 12 months, KSS-knee was 85.6 ± 4.1 (95% CI 84.5 to 86.8) in lim.-KA-II and 78.8 ± 3.2 (95% CI 77.9 to 79.7) in MA-II (*p* < 0.001). KSS-function followed the same pattern, reaching 91.8 ± 2.7 (95% CI 91.0 to 92.5) in lim.-KA-II and 87.8 ± 3.3 (95% CI 86.8 to 88.7) in MA-II (*p* < 0.001). These findings are compatible with an exploratory functional signal, but they should not be interpreted as confirmatory superiority (Table 3).

### 3.6. Patient-Reported Outcomes

The Oxford Knee Score (OKS) was similar between groups in the mild-varus subgroup. At 12 months, OKS was 45.4 ± 1.1 (95% CI 45.2 to 45.6) in lim.-KA-I and 45.6 ± 1.1 (95% CI 45.4 to 45.8) in MA-I (*p* = 0.194), suggesting no meaningful difference in short-term patient-perceived function when deformity severity was limited.

In the moderate-varus subgroup, OKS was numerically higher after lim.-KA across the postoperative course. The 12-month OKS was 45.3 ± 0.9 (95% CI 45.1 to 45.6) in lim.-KA-II and 38.9 ± 6.5 (95% CI 37.1 to 40.8) in MA-II (*p* < 0.001). FJS-12 also favored lim.-KA in the moderate-varus subgroup at 3 months (68.3 ± 3.6 versus 63.1 ± 7.3; *p* < 0.001), 6 months (82.6 ± 3.3 versus 78.2 ± 7.1; *p* = 0.001), and 12 months (90.5 ± 5.3 versus 79.3 ± 3.8; *p* < 0.001).

**Figure 2:**
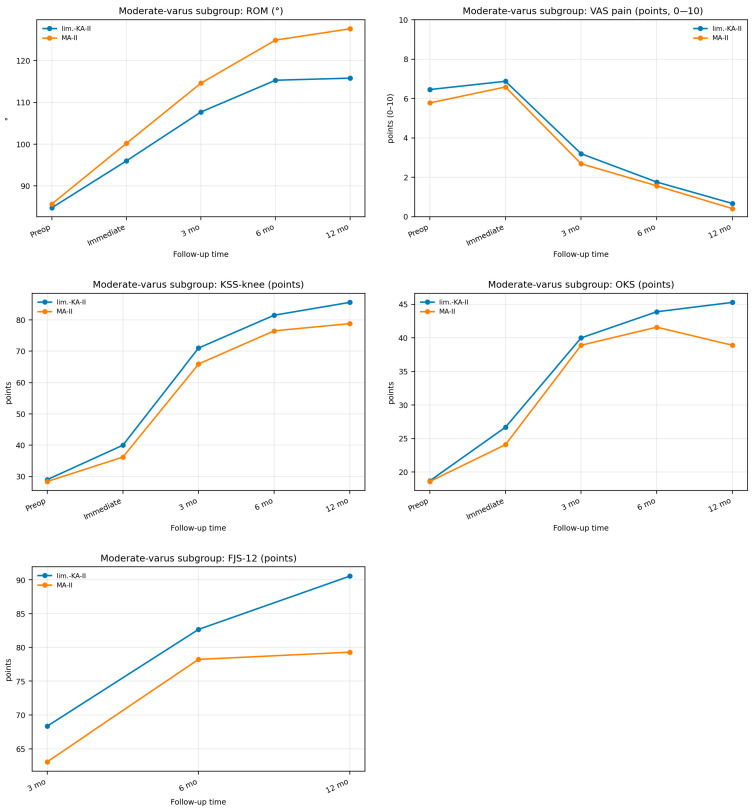
Unadjusted longitudinal trends in selected outcomes for the moderate-varus subgroup based on patient-level data. The longitudinal outcome patterns are shown descriptively in Table 3, and the 12-month unadjusted effect-size estimates for the moderate-varus subgroup are summarized in Table 4. °—degrees.

FJS-12 was similar between alignment strategies in the mild-varus subgroup at all available postoperative time points. In the moderate-varus subgroup, FJS-12 scores were higher after lim.-KA than after MA at 3, 6, and 12 months. At 3 months, FJS-12 was 68.33 ± 3.64 points in lim.-KA-II versus 63.09 ± 7.35 points in MA-II (*p* < 0.001). At 6 months, values were 82.65 ± 3.35 and 78.21 ± 7.09 points, respectively (*p* = 0.001). At 12 months, FJS-12 was 90.54 ± 5.26 in lim.-KA-II and 79.28 ± 3.76 in MA-II (*p* < 0.001). These findings indicate a consistent exploratory joint-awareness signal favoring lim.-KA in the moderate-varus subgroup (Figure 2, Table 3).

These PROM data identify an exploratory pattern in knees with greater preoperative varus and larger correction magnitude; they should be interpreted as a hypothesis-generating signal rather than definitive evidence that lim.-KA improves subjective joint integration (Table 3).

The principal 12-month unadjusted between-group effect-size estimates in the moderate-varus subgroup are summarized in Table 4.

The unadjusted longitudinal trajectories for selected outcomes in the moderate-varus subgroup are illustrated in Figure 2. The effect-size estimates and longitudinal plots are intended to complement the nominal *p*-values by showing the magnitude, direction, and precision of the exploratory subgroup differences. In the patient-level analysis, this pattern was not uniform across outcomes: KSS and PROMs favored lim.-KA in the moderate-varus subgroup, whereas ROM and VAS did not.

Exploratory, unadjusted, post hoc patient-level interaction analyses were then performed to assess whether the association between alignment strategy and outcome differed according to deformity severity (Table 5). At 12 months, the exploratory, unadjusted, post hoc alignment-by-deformity interaction term was statistically significant for ROM (−14.8°; 95% CI −20.9 to −8.7; *p* < 0.001), KSS-knee (+8.2 points; 95% CI 6.1 to 10.3; *p* < 0.001), KSS-function (+4.5 points; 95% CI 3.1 to 5.8; *p* < 0.001), OKS (+6.6 points; 95% CI 5.3 to 8.0; *p* < 0.001), and FJS-12 (+11.4 points; 95% CI 8.9 to 13.9; *p* < 0.001). The exploratory, unadjusted, post hoc interaction term was not statistically significant for VAS pain on the 0–10 scale (+0.14 points; 95% CI −0.09 to 0.37; *p* = 0.242). In repeated-measure sensitivity analysis using exploratory, unadjusted, post hoc generalized estimating equations, the time-varying alignment-by-deformity-by-time interaction was significant for ROM (*p* < 0.001), KSS-knee (*p* < 0.001), KSS-function (*p* < 0.001), OKS (*p* < 0.001), and FJS-12 (*p* < 0.001), but not for VAS pain (*p* = 0.145). These analyses support an exploratory, unadjusted, post hoc, outcome-specific interaction between alignment strategy and deformity severity, rather than a uniform benefit of one alignment strategy across all measured endpoints (Table 3, Table 4 and Table 5).

### 3.7. Safety Observations

No significant intraoperative or postoperative complications, such as intraoperative fracture, robotic-system failure requiring conversion to conventional instrumentation or manual completion of bone cuts, wound problems, hematoma, infection, thromboembolism, stiffness/manipulation under anesthesia, readmission, reoperation, neurologic or vascular events, were reported in the provided dataset. The complications were recorded retrospectively from the medical record, and any minor or transient adverse events that occurred were not classified as complications. Therefore, safety findings should be interpreted descriptively and not used to infer superiority or equivalence between the two alignment strategies.

## 4. Discussion

In this report, two findings must be separated. First, the primary whole-cohort randomized comparison did not show an overall advantage of lim.-KA over MA. Second, the exploratory, unadjusted, post hoc subgroup analysis suggested a possible phenotype-dependent pattern for KSS and PROMs, but did not prove a treatment-by-phenotype interaction across all outcomes. In knees with mild preoperative varus deformity, no meaningful or clinically consistent difference was observed between lim.-KA and MA during the first postoperative year. In contrast, in knees with moderate varus deformity requiring larger correction angles, patient-level analyses favored lim.-KA for KSS-knee, KSS-function, OKS, and FJS-12, but not for VAS pain, whereas ROM favored MA. Because these findings emerged only after post hoc subgroup stratification, no significant difference was observed in the full study cohort, and multiple repeated comparisons were performed without formal adjustment; the findings should be interpreted as exploratory and hypothesis-generating rather than as definitive evidence of treatment superiority.

The sustained ROM difference at 12 months is clinically relevant because it shows that the exploratory, unadjusted, post hoc interaction pattern was not uniform across endpoints. In the moderate-varus subgroup, ROM was higher after MA than after lim.-KA throughout postoperative follow-up, including at 12 months. This finding may reflect a trade-off between alignment target, correction magnitude, and motion recovery in knees requiring larger coronal correction. It also argues against a generalized claim that lim.-KA was superior in the moderate-varus phenotype. In this dataset, the moderate-varus signal favored lim.-KA for KSS and PROMs, whereas ROM favored MA, and pain did not favor lim.-KA. This discordant outcome pattern should be confirmed in prospective studies before being used to guide alignment selection.

This interpretation is biologically plausible but remains unproven. Varus knees are not uniform in their anatomy or soft-tissue behavior [10,11,12]. The more pronounced the deformity, the more likely it is that neutral mechanical correction requires a larger departure from the patient’s pre-arthritic joint-line orientation and constitutional alignment. Such correction may alter medial collateral ligament tension, lateral laxity, extension–flexion balance, and the amount of soft-tissue balancing required to obtain a stable knee. A bounded patient-specific strategy, such as lim.-KA, may preserve more of the native biomechanical envelope while remaining within acceptable safety limits [7,8,9]. The present data are compatible with that concept for clinician-derived scores and PROMs, but not for all outcomes. The ROM and VAS findings specifically argue against describing the moderate-varus subgroup result as a uniform clinical superiority of lim.-KA.

The radiographic findings help interpret the clinical data. Postoperative HKA values confirmed that lim.-KA and MA produced distinct coronal outcomes rather than minor variations around a shared target. In both mild and moderate varus, lim.-KA maintained a postoperative HKA around 176.7–176.9°, whereas MA restored HKA toward 179.1–179.3°. At the same time, the absence of meaningful clinical separation in the mild-varus subgroup suggests that the effect of these different radiographic endpoints is not uniform across all varus knees. In mild deformity, both strategies may result in final knee mechanics sufficiently similar for the patient to perceive equivalent short-term benefit.

The present findings are broadly compatible with current reports on TKA alignment methods. Network meta-analysis and updated meta-analyses have generally shown either similar overall outcomes between MA and kinematic/restricted-kinematic techniques or only modest short-term advantages for the kinematic family of alignments [25,26,27,28,29]. These reports are informative but also heterogeneous. If the true effect of alignment strategy depends on deformity severity, then averaging across knees with markedly different correction requirements may dilute a phenotype-specific signal. The current study offers a possible hypothesis-generating example: the overall randomized comparison was negative, whereas the moderate-varus subgroup showed an unadjusted exploratory pattern favoring lim.-KA for KSS and PROMs but not for ROM or VAS. This between-subgroup contrast must be tested formally in future work rather than inferred from subgroup *p*-values alone.

This interpretation is also consistent with recent work emphasizing balance and joint-line preservation rather than alignment labels alone. Restrictive kinematic protocols have been associated with improved quantitative soft-tissue balance compared with neutral mechanical strategies [30]. Functional or patient-specific alignment approaches have also been reported to achieve more balanced knees before soft-tissue release and to better restore joint-line obliquity than purely mechanical alignment in selected settings [31,32]. These studies do not prove that lim.-KA is universally superior, but they support a mechanistic framework in which patient-specific coronal targets may be most relevant when deformity is large enough for neutral correction to substantially alter native ligament–bone relationships.

The robotic aspect of the study is equally relevant. Robotic systems improve the fidelity with which planned alignment targets are executed [13,14,15]. That matters because individualized alignment concepts are only testable when the intended limb and component positions are reproduced accurately. In that sense, robotics is best understood here as an enabling technology rather than the treatment effect itself. This interpretation is compatible with meta-analytic evidence showing improved alignment precision with raTKA but only modest or inconsistent short-term clinical superiority over conventional instrumentation [16,17]. The present study, therefore, does not show that robotics alone improves pain or function; rather, it suggests that robotics may facilitate more faithful implementation of an alignment strategy whose effect depends on the phenotype and the magnitude of correction. That distinction is important when interpreting different arthroplasty studies, because technical platforms and alignment philosophies are often discussed together even though they address different questions: one concerns how accurately a plan can be executed, and the other concerns whether the chosen plan is biologically appropriate for a given knee.

Clinical relevance was considered alongside statistical significance. Published thresholds vary and are often derived for within-patient change rather than between-group differences, so they cannot be applied mechanically to this post hoc subgroup analysis [21,22,23,24,33]. In the moderate-varus subgroup, the OKS difference exceeded commonly reported OKS MCID/MIC thresholds, and the FJS-12 difference represented a substantial numerical separation that approached but did not clearly exceed the 1-year MIC reported in some cohorts. The KSS-knee difference exceeded some published MCID estimates but not all thresholds, whereas the KSS-function difference was smaller [21,22]. ROM and VAS did not support a lim.-KA advantage. The clinical interpretation should therefore be outcome-specific rather than a global claim of superiority.

Several limitations must restrict interpretation. The subgroup analysis was retrospective and post hoc, which substantially limits causal inference. The study was conducted at a single center in an open-label setting, and surgeon experience or local workflow may limit generalizability. Multiple outcomes were examined repeatedly over time without formal multiplicity correction. Although patient-level data allowed exploratory interaction and baseline-adjusted VAS analyses, these analyses were not prespecified and should be treated as sensitivity analyses rather than confirmatory tests. The moderate-varus subgroups differed significantly in baseline preoperative pain, which complicates interpretation of later VAS differences. Follow-up was limited to 12 months, precluding conclusions regarding implant survival, progressive radiographic change, aseptic loosening, or the long-term safety of different residual alignment targets.

The registered protocol and the present secondary manuscript address different levels of analysis. The registry lists CT-based implant-position measurements as the protocol-level primary outcomes and OKS as a secondary outcome, whereas the present manuscript is focused on clinical outcomes and uses 12-month OKS as the principal clinical endpoint for this secondary clinical-outcome analysis. In addition, the registry listed an estimated enrolment of 150 patients, while the available analytic dataset for the present analysis included 296 randomized patients. These differences are explicitly reported to clarify that the present manuscript should be interpreted as a secondary clinical-outcome analysis rather than as the primary registered imaging-outcome report.

Despite these limitations, the observed pattern is clinically relevant but should not be treated as practice-changing. The absence of group separation in mild deformity argues against indiscriminate adoption of lim.-KA for all varus knees based solely on short-term outcomes. Conversely, the coherent favorable signal in moderate deformity suggests a testable hypothesis: the deformity phenotype and the magnitude of correction may modify the clinical association between alignment strategy and outcome. The clinically useful threshold suggested by this dataset is best framed as an operational hypothesis: patients with preoperative varus >10° or correction requirements of approximately ≥10° may be the group in whom alignment target selection deserves focused prospective evaluation. This threshold is not validated and should not be used as a treatment rule outside a confirmatory study. Rather than asking whether MA or lim.-KA is globally better, future trials should ask which prespecified alignment target is best suited to which varus phenotype, using prospective stratification, patient-level longitudinal models, adjusted pain analyses, multiplicity-aware inference, and longer-term safety follow-up.

Future investigations should therefore be designed prospectively around phenotype. Studies should stratify patients by preoperative varus severity or constitutional alignment pattern before randomization, prespecify the role of correction magnitude, and use longitudinal repeated-measures models selected according to the estimation, such as mixed-effect models for subject-specific effects or generalized estimating equations for population-average effects. A prespecified interaction analysis should be included so that any claim of a phenotype-dependent treatment effect is supported by a direct statistical test rather than by comparison of separate subgroup *p*-values. Radiographic outlier analysis, soft-tissue balancing interventions, complications, and longer-term survivorship should be reported alongside PROMs. Such work is required to determine whether the present exploratory signal represents a true deformity-specific treatment effect.

## 5. Conclusions

The primary randomized comparison did not demonstrate an overall short-term clinical advantage of limited/restricted kinematic alignment over mechanical alignment in the full cohort. In post hoc exploratory analyses, mild varus deformity was associated with broadly similar outcomes after both alignment strategies. In the moderate-varus subgroup, patient-level analyses suggested more favorable KSS, OKS, and FJS-12 outcomes after lim.-KA, but ROM and VAS did not support a uniform clinical advantage. These findings should be interpreted as hypothesis-generating only and should not be used to change clinical practice without prospective, prespecified confirmation of an alignment-by-deformity interaction.

## Figures and Tables

**Table 1:** Baseline characteristics of deformity-matched subgroups. Values are mean ± standard deviation with 95% confidence intervals for the group mean. Within matched deformity strata, age, BMI, preoperative HKA, and preoperative ROM were similar. A baseline difference in VAS pain was observed in the moderate-varus subgroup (*p* = 0.026), which should be considered when interpreting subsequent pain comparisons.

Variable	lim.-KA-I (*n* = 99)	MA-I (*n* = 102)	lim.-KA-II (*n* = 48)	MA-II (*n* = 47)
Age, years	66.8 ± 7.1 95% CI 65.4 to 68.2	66.5 ± 7.7 95% CI 65.0 to 68.0	67.6 ± 7.4 95% CI 65.5 to 69.8	66.7 ± 7.0 95% CI 64.6 to 68.7
BMI, kg/m^2^	30.22 ± 3.52 95% CI 29.52 to 30.93	30.23 ± 3.97 95% CI 29.45 to 31.01	29.98 ± 3.73 95% CI 28.90 to 31.06	30.93 ± 3.09 95% CI 30.02 to 31.83
Preoperative HKA (degrees)	172.69 ± 1.03 95% CI 172.48 to 172.89	172.80 ± 1.06 95% CI 172.60 to 173.01	165.73 ± 2.96 95% CI 164.87 to 166.59	165.68 ± 2.75 95% CI 164.87 to 166.49
Preoperative ROM (degrees)	85.4 ± 4.7 95% CI 84.5 to 86.3	85.5 ± 4.7 95% CI 84.5 to 86.4	84.8 ± 5.5 95% CI 83.2 to 86.4	85.7 ± 3.9 95% CI 84.6 to 86.8
Preoperative VAS pain, points (0–10)	6.37 ± 1.49 95% CI 6.07 to 6.67	6.54 ± 1.41 95% CI 6.26 to 6.82	6.46 ± 1.37 95% CI 6.06 to 6.86	5.78 ± 1.49 95% CI 5.34 to 6.22

**Table 2:** Postoperative HKA and correction magnitude. Values are mean ± standard deviation with 95% confidence intervals for the group mean. The postoperative HKA values differed by alignment philosophy, consistent with the intended execution of lim.-KA versus MA. Within each method, postoperative HKA did not differ significantly between the mild and moderate deformity levels in the reported analysis.

Variable	lim.-KA-I (*n* = 99)	MA-I (*n* = 102)	lim.-KA-II (*n* = 48)	MA-II (*n* = 47)
Postoperative HKA (degrees)	176.88 ± 1.10 95% CI 176.66 to 177.10	179.28 ± 1.00 95% CI 179.09 to 179.48	176.73 ± 1.05 95% CI 176.43 to 177.03	179.09 ± 1.12 95% CI 178.76 to 179.41
Correction angle (degrees)	4.20 ± 1.44 95% CI 3.91 to 4.49	6.48 ± 1.48 95% CI 6.19 to 6.77	11.06 ± 3.10 95% CI 10.16 to 11.96	13.30 ± 2.87 95% CI 12.45 to 14.14

**Table 3:** Longitudinal clinical outcomes across follow-up in deformity-stratified subgroups. Values are mean ± standard deviation with 95% confidence intervals for the group mean. lim.-KA-I/MA-I: mild varus deformity (≤10°). lim.-KA-II/MA-II: moderate varus deformity (11–20°).

Outcome/Time Point	lim.-KA-I (*n* = 99)	MA-I (*n* = 102)	*p*	lim.-KA-II (*n* = 48)	MA-II (*n* = 47)	*p*
ROM, °						
Preoperative	85.4 ± 4.7 95% CI 84.5 to 86.3	85.5 ± 4.7 95% CI 84.5 to 86.4	0.889	84.8 ± 5.5 95% CI 83.2 to 86.3	85.7 ± 3.9 95% CI 84.6 to 86.8	0.768
Immediate postoperative	98.5 ± 7.2 95% CI 97.1 to 99.9	97.8 ± 6.8 95% CI 96.5 to 99.1	0.406	96.0 ± 7.0 95% CI 94.0 to 98.0	100.2 ± 7.0 95% CI 98.2 to 102.2	0.006
3 months	111.5 ± 8.9 95% CI 109.7 to 113.2	111.4 ± 8.7 95% CI 109.7 to 113.1	0.939	107.7 ± 9.7 95% CI 104.9 to 110.4	114.6 ± 6.5 95% CI 112.8 to 116.5	<0.001
6 months	118.2 ± 11.8 95% CI 115.9 to 120.5	115.4 ± 10.4 95% CI 113.4 to 117.4	0.066	115.3 ± 13.1 95% CI 111.6 to 119.0	124.9 ± 8.8 95% CI 122.3 to 127.4	<0.001
12 months	119.4 ± 13.2 95% CI 116.8 to 122.0	116.4 ± 12.4 95% CI 114.0 to 118.8	0.081	115.8 ± 14.5 95% CI 111.7 to 119.9	127.6 ± 8.1 95% CI 125.3 to 129.9	<0.001
VAS pain, points (0–10)						
Preoperative	6.37 ± 1.49 95% CI 6.08 to 6.67	6.54 ± 1.41 95% CI 6.27 to 6.81	0.348	6.46 ± 1.37 95% CI 6.07 to 6.85	5.78 ± 1.49 95% CI 5.35 to 6.20	0.026
Immediate postoperative	6.73 ± 1.61 95% CI 6.41 to 7.05	6.47 ± 1.48 95% CI 6.19 to 6.76	0.248	6.88 ± 1.13 95% CI 6.56 to 7.20	6.59 ± 1.92 95% CI 6.04 to 7.14	0.808
3 months	3.04 ± 0.92 95% CI 2.85 to 3.22	3.13 ± 0.93 95% CI 2.95 to 3.31	0.281	3.20 ± 0.84 95% CI 2.96 to 3.44	2.69 ± 0.90 95% CI 2.43 to 2.95	0.030
6 months	1.73 ± 0.57 95% CI 1.62 to 1.84	1.82 ± 0.58 95% CI 1.71 to 1.94	0.121	1.76 ± 0.53 95% CI 1.61 to 1.91	1.57 ± 0.51 95% CI 1.43 to 1.72	0.374
12 months	0.38 ± 0.50 95% CI 0.29 to 0.48	0.26 ± 0.40 95% CI 0.18 to 0.34	0.447	0.67 ± 0.55 95% CI 0.52 to 0.83	0.41 ± 0.52 95% CI 0.26 to 0.56	0.059
KSS-knee						
Preoperative	28.4 ± 4.5 95% CI 27.6 to 29.3	28.8 ± 4.7 95% CI 27.9 to 29.7	0.578	29.0 ± 4.1 95% CI 27.8 to 30.1	28.4 ± 4.5 95% CI 27.1 to 29.7	0.382
Immediate postoperative	42.3 ± 6.3 95% CI 41.1 to 43.6	44.4 ± 5.4 95% CI 43.3 to 45.4	0.021	40.0 ± 6.0 95% CI 38.3 to 41.7	36.2 ± 6.9 95% CI 34.3 to 38.2	0.010
3 months	72.2 ± 3.4 95% CI 71.5 to 72.8	72.9 ± 3.6 95% CI 72.2 to 73.6	0.131	71.0 ± 3.3 95% CI 70.1 to 71.9	65.9 ± 3.6 95% CI 64.9 to 66.9	<0.001
6 months	82.1 ± 3.3 95% CI 81.5 to 82.8	82.3 ± 3.5 95% CI 81.6 to 83.0	0.840	81.5 ± 3.4 95% CI 80.5 to 82.5	76.5 ± 4.0 95% CI 75.3 to 77.6	<0.001
12 months	86.3 ± 4.5 95% CI 85.4 to 87.2	87.7 ± 4.7 95% CI 86.7 to 88.6	0.032	85.6 ± 4.1 95% CI 84.5 to 86.8	78.8 ± 3.2 95% CI 77.9 to 79.7	<0.001
KSS-function						
Preoperative	31.8 ± 6.2 95% CI 30.6 to 33.1	30.8 ± 6.2 95% CI 29.6 to 32.0	0.205	30.9 ± 5.4 95% CI 29.3 to 32.4	31.7 ± 3.0 95% CI 30.8 to 32.5	0.060
Immediate postoperative	41.2 ± 4.9 95% CI 40.2 to 42.1	41.2 ± 5.2 95% CI 40.2 to 42.2	0.915	40.0 ± 4.3 95% CI 38.7 to 41.2	34.8 ± 2.4 95% CI 34.1 to 35.5	<0.001
3 months	79.6 ± 4.3 95% CI 78.8 to 80.5	80.8 ± 4.6 95% CI 79.9 to 81.7	0.078	78.1 ± 3.7 95% CI 77.0 to 79.1	74.0 ± 4.8 95% CI 72.7 to 75.4	<0.001
6 months	87.7 ± 3.2 95% CI 87.0 to 88.3	87.8 ± 3.0 95% CI 87.2 to 88.4	0.637	86.9 ± 3.2 95% CI 86.0 to 87.8	82.3 ± 3.4 95% CI 81.3 to 83.3	<0.001
12 months	92.5 ± 2.7 95% CI 92.0 to 93.0	93.0 ± 2.4 95% CI 92.5 to 93.4	0.248	91.8 ± 2.7 95% CI 91.0 to 92.5	87.8 ± 3.3 95% CI 86.8 to 88.7	<0.001
OKS						
Preoperative	19.3 ± 6.0 95% CI 18.2 to 20.5	20.2 ± 5.4 95% CI 19.2 to 21.3	0.315	18.7 ± 5.1 95% CI 17.2 to 20.1	18.6 ± 7.2 95% CI 16.5 to 20.7	0.952
Immediate postoperative	28.3 ± 4.2 95% CI 27.4 to 29.1	29.3 ± 4.0 95% CI 28.5 to 30.1	0.129	26.7 ± 3.6 95% CI 25.7 to 27.7	24.1 ± 3.1 95% CI 23.2 to 24.9	<0.001
3 months	40.6 ± 3.2 95% CI 40.0 to 41.3	41.1 ± 3.4 95% CI 40.5 to 41.8	0.347	40.0 ± 2.5 95% CI 39.3 to 40.8	38.9 ± 3.5 95% CI 37.9 to 39.8	0.338
6 months	44.9 ± 2.3 95% CI 44.4 to 45.3	45.4 ± 2.5 95% CI 44.9 to 45.8	0.145	43.9 ± 1.7 95% CI 43.4 to 44.3	41.6 ± 2.1 95% CI 41.0 to 42.1	<0.001
12 months	45.4 ± 1.1 95% CI 45.2 to 45.6	45.6 ± 1.1 95% CI 45.4 to 45.8	0.194	45.3 ± 0.9 95% CI 45.1 to 45.6	38.9 ± 6.5 95% CI 37.1 to 40.8	<0.001
FJS-12						
3 months	63.9 ± 6.9 95% CI 62.5 to 65.2	62.6 ± 7.3 95% CI 61.2 to 64.0	0.229	68.3 ± 3.6 95% CI 67.3 to 69.4	63.1 ± 7.3 95% CI 61.0 to 65.2	<0.001
6 months	79.0 ± 6.6 95% CI 77.7 to 80.3	77.9 ± 7.2 95% CI 76.5 to 79.3	0.339	82.6 ± 3.3 95% CI 81.7 to 83.6	78.2 ± 7.1 95% CI 76.2 to 80.2	0.001
12 months	91.3 ± 5.3 95% CI 90.3 to 92.3	91.5 ± 5.5 95% CI 90.4 to 92.5	0.711	90.5 ± 5.3 95% CI 89.1 to 92.0	79.3 ± 3.8 95% CI 78.2 to 80.4	<0.001

**Table 4:** Key 12-month unadjusted effect-size estimates in the moderate-varus subgroup. Mean differences are calculated as lim.-KA-II minus MA-II using patient-level data. Positive values favor lim.-KA for KSS, OKS, and FJS-12; negative values favor MA for ROM; positive values for VAS indicate higher pain after lim.-KA on the 0–10 scale. These estimates are exploratory and unadjusted for multiplicity; VAS is additionally affected by baseline pain imbalance.

Outcome	Mean Difference	95% CI	Clinical Interpretation
ROM, °	−11.8	−16.6 to −7.0	Unadjusted difference favored MA rather than lim.-KA; ROM does not support a lim.-KA advantage.
VAS pain, points (0–10)	+0.26	0.05 to 0.48	Higher pain after lim.-KA descriptively; baseline-adjusted analysis did not support a lim.-KA pain advantage.
KSS-knee	+6.8	5.3 to 8.3	Magnitude exceeds the KSS-knee MCID range reported by Lee et al. but remains below the more conservative threshold reported by Lizaur-Utrilla et al. [21,22].
KSS-function	+4.0	2.7 to 5.2	Statistically separated, but the 4.0-point difference is below published MCID thresholds for the KSS-function domain [21,22].
OKS	+6.4	4.5 to 8.3	Likely clinically relevant because the 6.4-point OKS difference exceeds commonly reported OKS MCID/MIC thresholds after TKA [23,24].
FJS-12	+11.3	9.4 to 13.1	Substantial numerical difference; the 11.3-point FJS-12 difference approaches but does not clearly exceed the 1-year MIC reported by Ingelsrud et al. [24].

**Table 5:** Exploratory, unadjusted, post hoc interaction analyses for alignment strategy × deformity severity. All interaction analyses are exploratory, unadjusted, post hoc analyses and should be interpreted descriptively.

Outcome	12-Month Exploratory, Unadjusted, Post Hoc Alignment × Deformity Interaction Estimate	Exploratory, Unadjusted, Post Hoc GEE Time-Varying Interaction *p*	Interpretation
ROM	−14.8° (95% CI −20.9 to −8.7; *p* < 0.001)	*p* < 0.001	Exploratory, unadjusted, post hoc significant interaction; the moderate-varus ROM pattern favored MA, not lim.-KA.
VAS pain	+0.14 points (95% CI −0.09 to 0.37; *p* = 0.242)	*p* = 0.145	Exploratory, unadjusted, post hoc 12-month interaction was not statistically significant; pain interpretation remains limited by baseline VAS imbalance.
KSS-knee	+8.2 points (95% CI 6.1 to 10.3; *p* < 0.001)	*p* < 0.001	Exploratory, unadjusted, post hoc significant interaction; supports an outcome-specific phenotype-dependent clinical-score signal favoring lim.-KA.
KSS-function	+4.5 points (95% CI 3.1 to 5.8; *p* < 0.001)	*p* < 0.001	Exploratory, unadjusted, post hoc significant interaction; supports an outcome-specific phenotype-dependent functional-score signal favoring lim.-KA.
OKS	+6.6 points (95% CI 5.3 to 8.0; *p* < 0.001)	*p* < 0.001	Exploratory, unadjusted, post hoc significant interaction; supports an outcome-specific phenotype-dependent PROM signal favoring lim.-KA.
FJS-12	+11.4 points (95% CI 8.9 to 13.9; *p* < 0.001)	*p* < 0.001	Exploratory, unadjusted, post hoc significant interaction; supports an outcome-specific phenotype-dependent joint-awareness signal favoring lim.-KA.

Abbreviations: GEE, generalized estimating equations; ROM, range of motion; VAS, visual analog scale; KSS, Knee Society Score; OKS, Oxford Knee Score; FJS-12, Forgotten Joint Score-12; PROM, patient-reported outcome measure. Interaction estimates are exploratory, unadjusted, post hoc patient-level model estimates. The GEE column reports the global time-varying alignment × deformity × time interaction *p*-value for the repeated-measure sensitivity analysis.

## Data Availability

The original contributions presented in this study are included in the article. Further inquiries can be directed to the corresponding author.
